# Commercialism and Charity

**Published:** 1903-10

**Authors:** 


### Commercialism and Charity.

THE newspapers are passing along a semi-humorous paragraph to the effect that a Pennsylvania physician has burned up as worthless $12,000 worth of accounts. The comments on this tale, true or otherwise, are various, and while there is an evident intent to make humor of it there is underlying a serious strain which amounts almost to pathos. The *Buffalo Express* in referring to the matter said that physicians were the only class of men in business who would permit that amount of accounts to stand long enough on their books to become "no good." That appears to be the keynote of all newspaper comment. Physicians are notably lax in business methods and suffer thereby.

Time and custom have made it a joke among those outside the profession that a doctor is always the last to be paid; and yet it speaks well for the profession that there are so few judgments entered against physicians in the courts of law for nonpayment of debt. The public knows nothing of the worries of professional life, of the struggle to collect accounts, the constant hammering of delinquents and the constant and callous evasion on the part of the once grateful debtor.

From a purely commercial standpoint, the average physician is a poor business man. The grocer, the butcher and the candlestick maker, when their bills are not paid promptly, cut off supplies to the delinquent family until they pay. If they don't liquidate the debt they must go somewhere else to trade. It lasts just long enough for the new dealer to learn through the commercial exchange system, which is in vogue in almost every branch of purely commercial business nowadays, that the new customer is a delinquent, and he is then asked to pay as he goes. On the other hand, if a physician does muster up sufficient courage to refuse his services to a family which may properly be classed under the "dead beat" list, another member of the profession will attend that family and continue to give them his time, his skill and his best endeavor without in the least attempting to find out if they are good for his bill when he presents it. He has no means of knowing whether the family is good pay, bad pay or no pay at all, because there is no system. Professional jealousy may account for this; perhaps something else may, but the condition exists nevertheless, and physicians will continue to carry a mass of wholly worthless accounts on their books until they make some effort to regulate the evil.

Nearly every city of any size employs district physicians whose work it is to care for the poor unable to pay for medical attendance. Where the poverty is so acute as to demand it, the city pays for the necessary medicines. Municipalities supply or pay for antitoxin and vaccine, and the careless physician gives his time to the administering of the drugs.

It is well and eminently proper that we should as a profession practise charity and relieve distress, heal the sick and comfort the afflicted; but it is not right that we should give away our best efforts to an unappreciative class of patients who do not pay, although they are able to, and who will not pay, although they can. Various suggestions have been made looking to a regulation of the evil of free services, but as yet none seems to have been advanced which is practical.

The little half-humorous paragraph which is going the rounds of the newspapers might furnish food for serious discussion at the meetings of county societies.

---

Walter Reed Memorial Fund.—On the 15th of August a meeting was held in Bar Harbor of friends of the late Major Reed, M. D., U. S. A., to whom in a large degree is due the discovery of the mode by which yellow fever has been spread and the consequent suppression of that dire disease. Representative men were present from distant parts of the country and letters were received from various members of committees already appointed to promote the collection of a memorial fund in grateful commemoration of Dr. Reed's services. Important suggestions were presented from President Eliot, Dr. W. W. Keen, Professor J. W. Mallet and others. Dr. Daniel C. Gilman, chairman of a committee appointed by the American Association for the Advancement of Science, presided, and Dr. Stuart Paton acted as secretary. Among those who took part in the conference were Dr. W. H. Welch, of Baltimore; Dr. Janeway, of New York; Dr. Abbott, of Philadelphia; Dr. Herter, of New York; Dr. Barker, of Chicago; Dr. Sajous, of Philadelphia; Dr. Putnam, of Buffalo, and Dr. Fremont Smith, of Bar Harbor; and also Bishop Lawrence, of Massachusetts, and Messrs. Morris K. Jesup, president of the New York Chamber of Commerce; John S. Kennedy, president of the Presbyterian Hospital of New York, and William J. Schieffelin, of New York.

The following conclusions were reached: that an effort should be made to raise a memorial fund of $25,000 or more, the income to be given to the widow and daughter of Dr. Reed, and after their decease the principal to be appropriated either to the promotion of researches in Dr. Reed's special field, or to the erection of a memorial in his honor at Washington.

Arrangements were made for the publication of circulars explaining this movement, and asking coöperation not only from the medical profession, but from all liberally disposed individuals who appreciate the value of Dr. Reed's services to mankind.

---

THE agitation toward a suppression of the toy pistol evil is becoming widespread and Buffalo has entered the lists and taken a firm stand against this apparently innocent and patriotic toy. It is the intention to not alone stop the sale of the blank cartridge pistol, but to prevent its use at any time. This is the only safe course. So long as there is a pistol available, the fearless and irrepressible small boy will get it; and if he cannot discharge it in the street he will go somewhere where he can. The small boy is naturally brave, but bravest when he is eating forbidden fruit. The long and constantly growing mortality list due to tetanus directly traceable to the use of the toy pistol has at last attracted the attention of the ordinarily complacent municipal legislators, and they are beginning to see that there is something in what the medical profession has been crying against for years, and they are taking action against it. It was the same way with diphtheria and the use of antitoxin. In some cities the suggestion to provide free antitoxin was granted readily enough, not because it was for the good of humanity alone, but because some of the able and far-seeing politicians saw "something in it." They saw the Mecca of all political life—graft! In other places where the possibilities were not seen or where they were unattainable, the plan was shelved until the matter was brought home to the objectors possibly by a death in their own families from the disease. Then with almost feverish haste was the plan adopted. There is no graft in the toy pistol trade; at least none for municipal politicians, and so the advice of science is listened to and followed. The wiping out of the toy pistol will save many lives and it is a gratification that Buffalo is taking such active and rigorous steps to prevent even the innocent possession of the dangerous article.

The third fascicle of contributions from the William Pepper laboratory of clinical medicine has been sent out within the last few weeks. It contains a group of papers reprinted from various magazines, bound in paper and issued in quarto form. A large portion of the book is devoted to the pathology of diseases of the nervous system by Spiller, Frazier, McCarthy and Ravenel. Other contributions relate to pancreatic disease, creatinin secretion, diabetes mellitus, urinary specific gravity and nitrogen elimination in pregnancy, postoperative changes in the blood, myositis fibrosa, granular degeneration of the erythrocyte, sarcoma of the large intestine, carcinoma of the eyelids, papilloma of the caruncle, and the surgical treatment of sterility, due to obstruction at the epididymis. It is a valuable brochure, but its convenience of reference would have been enhanced very much if it were paged consecutively and indexed.

---

It has remained for an enterprising British insurance corporation to take the lead in bizarre risks and steal a march on the boasted enterprise of the Yankee. The Royal Exchange Assurance Company, of England, has sent out a prospectus, offering to insure against appendicitis at the rate of $1.25 a year per each $500 of insurance. The policy guarantees the holder against medical, surgical and nursing expenses up to the amount insured for. *The Lancet* has taken the matter up and in a tone which, when one considers the usually serious import of *Lancet* utterances, might be said to be almost flippant in tone, the famous medical journal wonders how the applicant can answer the question: "Have you or your family ever suffered from appendicitis, or from any of the symptoms pertaining to it?" *American Medicine* has a say, too, in a recent issue and thus cleverly handles the subject:

What is meant by "family," and is a pain in the belly a symptom of this disease only? Moreover, has the patient the requisite medical knowledge either of himself or his family to give a discriminating answer? The Insurance Company's leaflet says that during 1900, 15,000 operations were performed in the United Kingdom for appendicitis. Were there so many in the whole world? The company estimates that about one in 400 per annum will be attacked by the disease. But would the rate be the same in the United States with its appendiceal beliefs as in conservative England? And then how about all the other ailments and accidents which may happen to one? There are a thousand ways in which one may be sick or die; should the prudent man not secure a policy for each one of them? This would in time result in a distinct form of monomania, a morbophobia which might be called insurance disease. Could the companies devise a policy for these afflicted ones?

---

THE osteopaths are beginning to sit up and notice one another in the columns of their official organs and what has been looked for is come to pass. They are beginning to find fault with one another as to the ethics of their "profession," and are drawing fine lines of distinction as between "regular" osteopaths and "lesion" osteopaths, whatever those terms may mean to the practitioners of massage.

The heretics believe there are other agencies than manipulation which will cure disease conditions and so the squabble has begun. In discussing the matter a "Dr. C. W. Young, D. O." says, referring to "Dr." Still's textbook on osteopathy, and be it remembered that "Dr." Still is the inventor of this method of massage:

Chapter XIV. is devoted to the treatment of diphtheria and other diseases by pouring glycerine and water in the ear. He described a desperate case of croup, where water, glycerine and a wet rag were used and "osteopathic treatment." I have heard the book ridiculed for this chapter. I do not believe in ridiculing anything until you know what you are talking about. I tried this ear treatment in the case of the surgically maimed child who died while under my treatment of diphtheria. It seemed to give relief and she begged to have it repeated. She seemed to be improving at the time she choked to death when the nurse attempted to force her to swallow too much water all at once. I am satisfied that this ear treatment is all right as well as all other treatments described by Dr. Still. Too many osteopaths are neglecting to read the Old Doctor's books. He says God and experience are his authorities. I believe in these authorities. They are far ahead of the authority of theory.

Which is chiefly interesting because it is so foolish.

---

THE state board of examiners of registered nurses, composed of L. Bissell Sandford, of New York; Miss Annie Damer, of Buffalo; Miss Dorothy N. McDonald, of Brooklyn; Miss Sophia F. Palmer, of Rochester, and Miss Jane Elizabeth Hitchcock, of New York, held a meeting September 15, 1903, and elected Miss Palmer, president, and Miss Hitchcock, secretary. Miss Palmer is editor of the *American Journal of Nursing*. Miss Hitchcock has charge of the nursing staff in the nurses' settlement, New York.

The test in practical nursing to be applied by the state board will include both a practical demonstration and a written test.

The practical demonstration will be conducted by a member of the board of examiners who must recommend the applicant for admission to the written test. Both practical demonstrations and written tests will be held on the dates and at the places prescribed for regents' examinations in the other professions.

---

GYNECOLOGY as a specialty is a goner. Russell Bellamy says so over his printed signature. Lest there be some unenlightened person that may inquire,—"And pray, who is Russell Bellamy"?—we remark that he is the associate editor of the *Medical Critic*, in charge of the department of gynecology. So, that appears to settle it; hence, we expect R. B. will seek other employment.

---

YELLOW fever is epidemic at Tampico and the health authorities are endeavoring to stamp out the disease. Over 200 residences which had been inhabited by yellow fever patients have been burned. Fire is naturally the best disinfectant, but such wholesale conflagration partakes more of the nature of pyromania than of science.

---

TEXAS, with past experiences with yellowjack in mind is taking precautionary measures against the epidemic which is raging in Mexico, by establishing fumigating plants along the Rio Grande, and the state health department has sent Dr. I. P. Seison to Monterey to watch the progress of the disease. In Mexican districts along the border, especially in Linares, panic prevails, and in the latter place, the population of which was 9,000, there are eight deaths a day. Half of the population has either died or fled.

---

*American Medicine* calls attention to what it terms a negro population paradox. Official figures are quoted to show that while the negro death-rate in cities exceeds the birth-rate, the negro population in the rural districts is increasing very rapidly; so rapidly in fact as to more than offset the decline in the cities. And bulking the negro population the official census figures show that there has been an increase of over 1,000,000 in each decade.

